# Jamestown Canyon virus in Massachusetts: clinical case series and vector screening

**DOI:** 10.1080/22221751.2020.1756697

**Published:** 2020-05-13

**Authors:** Cormac M. Kinsella, Molly L. Paras, Sandra Smole, Samar Mehta, Vijay Ganesh, Lin H. Chen, Daniel P. McQuillen, Ruta Shah, Justin Chan, Matthew Osborne, Scott Hennigan, Frederic Halpern-Smith, Catherine M. Brown, Pardis Sabeti, Anne Piantadosi

**Affiliations:** a Infectious Disease and Microbiome Program, Broad Institute of MIT and Harvard, Cambridge, MA, USA; b Department of Organismic and Evolutionary Biology, Harvard University, Cambridge, MA, USA; c Department of Medicine, Division of Infectious Diseases, Massachusetts General Hospital, Boston, MA, USA; d Harvard Medical School, Boston, MA, USA; e Bureau of Infectious Disease and Laboratory Sciences, Massachusetts Department of Public Health, Jamaica Plain, MA, USA; f Department of Medicine, Division of Infectious Diseases, Beth Israel Deaconess Medical Center, Boston, MA, USA; g Department of Neurology, Massachusetts General Hospital, Boston, MA, USA; h Department of Medicine, Division of Infectious Diseases and Travel Medicine, Beth Israel Lahey Health, Mount Auburn Hospital, Cambridge, MA, USA; i Department of Infectious Disease, Beth Israel Lahey Health, Lahey Hospital and Medical Center, Burlington, MA, USA; j Department of Medicine, Division of Geographic Medicine and Infectious Diseases, Tufts University School of Medicine, Boston, MA, USA; k Department of Medicine, Division of Infectious Diseases, North Shore Medical Center, Salem, MA, USA; l Department of Immunology and Infectious Disease, Harvard T.H. Chan School of Public Health, Cambridge, MA, USA; m Howard Hughes Medical Institute, Chevy Chase, MD, USA

**Keywords:** Jamestown Canyon virus, arbovirus, encephalitis, meningitis, diagnostics

## Abstract

Jamestown Canyon virus (JCV) is a neuroinvasive arbovirus that is found throughout North America and increasingly recognized as a public health concern. From 2004 to 2012, an average of 1.7 confirmed cases were reported annually in the United States, whereas from 2013 to 2018 this figure increased over seventeen-fold to 29.2 cases per year. The rising number of reported human infections highlights the need for better understanding of the clinical manifestations and epidemiology of JCV. Here, we describe nine patients diagnosed with neuroinvasive JCV infection in Massachusetts from 2013, the year of the first reported case in the state, to 2017. Because current diagnostic testing relies on serology, which is complicated by cross-reactivity with related orthobunyaviruses and can be negative in immunosuppressed patients, we developed and evaluated an RT-qPCR assay for detection of JCV RNA. We tested this on the available archived serum from two patients, but did not detect viral RNA. JCV is transmitted by multiple mosquito species and its primary vector in Massachusetts is unknown, so we additionally applied the RT-qPCR assay and confirmatory RNA sequencing to assess JCV prevalence in a vector candidate, *Ochlerotatus canadensis*. We identified JCV in 0.6% of mosquito pools, a similar prevalence to neighboring Connecticut. We assembled the first Massachusetts JCV genome directly from a mosquito sample, finding high identity to JCV isolates collected over a 60-year period. Further studies are needed to reconcile the low vector prevalence and low rate of viral evolutionary change with the increasing number of reported cases.

## Introduction

Jamestown Canyon virus (JCV) is an arbovirus in the genus *Orthobunyavirus* that can cause acute febrile illness, severe meningitis, and encephalitis [1]. An RNA virus with a segmented negative sense genome, JCV was first isolated in Colorado in 1961 and is limited to but widely distributed across North America, where it circulates between mosquitoes and its principal reservoir, white-tailed deer. Human JCV infection was first described in 1980 [2]; it became reportable in 2004, and only 15 cases were reported through 2012 [1,3]. However, after the Centers for Disease Control and Prevention (CDC) introduced routine JCV testing for suspected domestic arboviral cases in 2013, 175 cases were reported from 2013 to 2018, including 75 in 2017 alone [4–9]. Approximately 60% of cases were neuroinvasive and three were fatal (1.7%). JCV infection likely remains under-recognized, with studies demonstrating seroprevalence in the range of 15–30% [10,11], and up to 54% (among 121 Alaskan reindeer herders) [12]. Mortality is rare [13] and may be related to sequelae from extended hospitalization [14], however, patients often have prolonged morbidity [3,15]. To characterize the clinical manifestations and outcome of JCV infection among patients diagnosed after the introduction of routine testing in Massachusetts, where the first case was reported in 2013, we abstracted data from nine patients between 2013 and 2017. Laboratory diagnosis of JCV is made by serology, but due to cross-reactivity with other arboviruses, diagnosis requires a screening antibody-capture ELISA and a confirmatory JCV-specific plaque reduction neutralization test (PRNT) [16]. Serology can be negative in patients treated with B-cell depleting agents, an increasingly recognized limitation for both JCV (Solomon *et al*., in preparation) and other arboviruses [17–19]. To assess the utility of viral RNA detection for JCV diagnostics, we designed a JCV RT-qPCR assay and screened available residual sera.

The primary vector of JCV in Massachusetts is unknown, with only three isolations in the west of the state reported in the literature, from *Aedes intrudens* and *Ochlerotatus abserratus* mosquitoes [20]. JCV has an exceptionally broad range of potential vectors, having been isolated from 26 mosquito and 3 tabanid fly species [21], with vector competency confirmed for 11 mosquito species [22–24]. A study in neighboring Connecticut identified JCV in *Ochlerotatus canadensis* in 40 of 91 locations and 9 of 10 years; JCV isolations were also made from this species with equal distribution across the state despite varying land use, suggesting that it is a primary local vector [21]. To address the hypothesis that this vector is similarly important in Massachusetts, we used our RT-qPCR assay combined with follow up RNA sequencing to investigate the prevalence of JCV in 359 *O. canadensis* pools containing 13,779 adult mosquitoes collected in Massachusetts from 2012 to 2016.

## Materials and methods

### Patient data and samples

Cases of JCV infection in Massachusetts between 2013 and 2017 were identified by the Massachusetts Department of Public Health (MDPH): this yielded nine cases from seven hospitals, and search of clinical records identified no additional cases. Clinical, laboratory, and imaging data were extracted from medical records. All patients were diagnosed by JCV-specific capture ELISA and PRNT using standard testing algorithms at the CDC. Archived serum was available for RT-qPCR testing from Patients 2 and 4, while acute serum and cerebrospinal fluid (CSF) from Patient 7 was tested by metagenomic sequencing as part of a separate study (Piantadosi *et al*., in preparation). The work was approved by the Institutional Review Board (IRB) under MDPH protocol 965856 and Partners protocol 2018P001282 (Mount Auburn Hospital ceded review and relied on Partners IRB for regulatory review; Beth Israel Deaconess Medical Center ceded review under protocol 2018C000630; Lahey Hospital made a determination of IRB exempt).

### Mosquito samples

Mosquito trapping with CDC light and CO_2_ traps was carried out annually (May through June) from 2012 to 2016 by the MDPH and collaborating mosquito control programs. Traps were set on average every 2 days, often in multiple localities, and traps were collected the following day. Mosquitoes were stored at 4°C, taxonomically sorted, and pooled (≤50 per pool). *O. canadensis* pools (*N* = 359) were homogenized in BA-1 medium, and total nucleic acid was extracted from 140 µl of homogenate using the MagNA Pure 96 DNA and Viral Nucleic Acid Small Volume kit (Roche, Basel, Switzerland). An aliquot was confirmed negative for West Nile virus (WNV) and Eastern equine encephalitis virus (EEE) by RT–PCR [25]. As a positive control, RNA was extracted from a commercially-available JCV culture supernatant (ATCC^®^ VR-712™, lot #12507) using MagNA Pure Total Nucleic Acid I (Roche, Basel, Switzerland). To verify non-viability, extracted material was inoculated onto Vero cells grown at 37°C in BA-1 medium (Supplementary Data), subpassaged once and monitored for cytopathogenic effects for 14 days.

### JCV RT-qPCR

JCV has a segmented (-)ssRNA genome: the S (small, ∼1 kb) segment encodes the nucleocapsid; M (medium, ∼4.5 kb) encodes the surface glycoprotein; and L (large, ∼7 kb) encodes the RNA-dependent RNA polymerase. S is the most sampled genome segment, with 109 published sequences at the time of writing (compared to under 10 each from M and L). We designed JCV-specific PCR primers within S by aligning published JCV sequences with the closely related Inkoo virus (IV) from Northern Europe, the synonymous Jerry Slough virus (JSV) and South River virus (SRV), and 6 more distantly related orthobunyaviruses using ClustalW (Supplementary Data). The primers target a 120 bp region: F: 5′-GCAGGGTTTGTGGCATTTATGG-3′, R: 5′-TTTCCGCTCCGGTTTACGAG-3′. DNA standards were constructed spanning the target (IDT, Coralville, IA, USA). RT-qPCR was carried out using the Power SYBR^®^ Green RNA-to-C_T_™ 1-Step kit (Thermo Fisher Scientific, Waltham, MA, USA), with 500 nM primers and 3 µl template in a 10 µl reaction. Conditions were: 48°C for 30 min, 95°C for 10 min, 45 cycles of 95°C for 15 s and 60°C for 30 s, and melting with 95°C for 15 s, 55°C for 15 s, 95°C for 15 s.

The RT-qPCR assay was validated on the positive control RNA, and products were analyzed for size and primer dimerization by TapeStation. RNA isolated from study samples – including archived serum samples from Patients 2 and 4 and all 359 mosquito pools – were screened in triplicate alongside standards, positive control RNA, and no template controls (NTCs). Because SYBR Green dye binds dsDNA nonspecifically, nonspecific amplification can produce false positive signals at higher PCR cycles. This necessitates the use of stringent negative controls. For this assay, out of 75 NTC wells, nonspecific amplification was detected in 2 (2.7%), with Ct values of 33.9 and 37.2. Therefore, for a sample to be called positive, we required amplification in all three wells, with an average Ct of <33.9. However, some mosquito samples close to this threshold were selected for further screening by RNA sequencing in order to ensure we did not miss true positive samples with low levels of viral RNA.

### RNA sequencing

Eleven of 359 mosquito pools underwent RNA sequencing: one that was JCV RT-qPCR positive, nine with negative RT-qPCR results close to the threshold (low-level amplification in ≥1 well), and one negative control pool. Samples were treated with TURBO™ DNase (Thermo Fisher Scientific, Waltham, MA, USA) and cleaned using Agencourt RNAClean XP beads (Beckman Coulter, Brea, CA, USA). Unbiased cDNA synthesis with random hexamer primers and SuperScript^®^ III (Thermo Fisher Scientific, Waltham, MA, USA) was followed by Nextera XT (Illumina, San Diego, CA, USA) fragmentation and 18-cycle PCR amplification. Libraries were quantified (KAPA Biosystems, Wilmington, MA, USA) and pooled at equimolar concentration. Sequencing was performed with MiSeq or HiSeq instruments (Illumina, San Diego, CA, USA) to obtain 150 bp or 100 bp paired-end reads. If multiple JCV reads were identified in a sample, an independent library was constructed from cDNA and sequenced for confirmation.

### JCV sequence analysis

Reads were processed using viral-ngs [26] (Supplementary Data). Human reads and contaminants were depleted before quality trimming, de-duplication, and filtering against a JCV sequence database (Supplementary Data). *De novo* assembly of filtered reads was attempted, followed by reference-assisted improvement. For samples with too few JCV reads to assemble contigs, reads were BLAST queried to confirm their identity. To assess the possibility of contamination between samples, reads underwent manual comparison to positive samples in this study, the positive control strain, and public JCV sequences.

Full-length JCV coding sequences were downloaded from GenBank in March 2019: 108 S sequences, 7 M sequences, and 6 L sequences, all from mosquito isolates. To these were added one JCV genome assembled in the course of this study, representative sequences from JSV, SRV, IV, and La Crosse virus (LACV) as an outgroup (Supplementary Data). Sequences were de-duplicated using CD-HIT [27], aligned with MAFFT (G-INS-I) [28], and trimmed using the strict setting of trimAl v1.3 [29]. Maximum likelihood phylogenetic trees were constructed using RAxML v8.2.9 with the GTRCAT substitution model and 200 bootstraps [30].

## Results

### JCV cases

Nine cases of JCV infection diagnosed by serology were identified in Massachusetts between 2013 and 2017. Their clinical presentations, detailed below and summarized in Supplementary Table 1, shared several notable features. Most patients were generally healthy, or only mildly immunocompromised, and had a high degree of outdoor exposure. Although no patients died, nearly all had a prolonged course including fatigue, headache, altered mental status, and other neurological symptoms.

*Patient 1*: A 57-year-old man presented with rash, myalgia, fatigue, and chills in July 2013. IgM Western blot was positive for *Borrelia burgdorferi*, and he was treated with doxycycline. Two days later, he developed impaired orientation, tangential thought with confabulation, and visual hallucinations. He had general myoclonus, bilateral asterixis and cogwheeling, and pharyngeal weakness from multiple cranial neuropathies. Brain MRI, obtained after lumbar puncture (LP), showed diffuse dural enhancement and chronic subdural collections. Analysis of CSF revealed lymphocytic pleocytosis with elevated protein (Table 1). The patient had medical comorbidities including diabetes but was not overtly immunocompromised. He lived in central Massachusetts and vacationed on Cape Cod. He had positive IgM for *Babesia microti* (PCR negative), and was treated for babesiosis and presumed Lyme meningoencephalitis. Ultimately, he was diagnosed with JCV, with positive IgM ELISA and a PRNT result of 1:160 (Table 2). He slowly improved during his one-month hospitalization, but memory deficits persisted for several months.

**Table 1.** Results of cerebrospinal fluid analysis from the patients described, except Patients 3 and 4 who did not undergo lumbar puncture.

	Patient 1	Patient 2	Patient 5	Patient 6	Patient 7	Patient 8	Patient 9
Time since symptom onset	<1 week	3 weeks	3 weeks	5 months	<1 week	2 weeks	<1 week
RBC*, tube 1 → 4	NR*	35 → 51	30 → 3	21 → 1	1298 → 8	0 → 0	1179 → 48
WBC*, tube 1 → 4	NR → 45	73 → 41	7 → 14	3 → 0	16 → 38	0 → 0	1 → 0
% lymphocytes	92	97	80	88	66		
CSF* glucose	NR	53	119	68	64	56	56
Peripheral glucose	NR	65	227	NR	114	94	108
CSF total protein (mg/dL)	104	112	76	38	78	30	47

*RBC = red blood cell (cells/mm^3^), WBC = white blood cell (cells/mm^3^), NR = not reported, CSF = cerebrospinal fluid.

**Table 2.** Results of Jamestown Canyon virus and other arbovirus tests from the patients described, blanks indicate no testing.

Assay	Sample	Patient 1	Patient 2	Patient 3	Patient 4	Patient 5	Patient 6	Patient 7	Patient 8	Patient 9
Time since symptom onset		< 1week	3 weeks	10 weeks	3 days	3 weeks	5 months	<1 week	2 weeks	<1 week
JCV* IgM ELISA^†^	Serum	+		+	+	+	+	+	+	+
JCV PRNT^†^	Serum	1:160		1:1280	1:320	1:160	1:80	1:2560	1:160	1:160
JCV IgM ELISA	CSF*		+						–	
JCV PRNT	CSF		1:1280						–	
WNV* IgM EIA^†^	Serum	–	–	–	–	–	Indeterminate	–	–	–
WNV PRNT	Serum						–	–		
WNV IgM EIA	CSF		–	–		–			–	
EEE* IgM EIA	Serum	–	–	–	–	–	–			–
EEE IgM EIA	CSF		–	–		–				
LACV* ELISA	Serum			+	+			Equivocal		–
LACV PRNT	Serum			1:160	1:160	1:20		1:40	–	
Powassan IgM ELISA	Serum		–	–		–	–	–	–	–
Powassan IgM ELISA	CSF								–	
SLEV* IgM IF^†^	Serum								≥1:10^‡^	
SLEV IgG IF	Serum								–	
SLEV/WNV IgM MIA^†^									–	

*JCV = Jamestown Canyon virus, WNV = West Nile virus, EEE = Eastern equine encephalitis virus, LACV = La Crosse virus, SLEV = Saint Louis encephalitis virus, CSF = cerebrospinal fluid.

^†^ELISA = enzyme-linked immunosorbent assay, PRNT = plaque reduction neutralization test, EIA = enzyme immunoassay, IF = immunofluorescence, MIA = microsphere immunoassay.

^‡^This finding was not replicated on confirmatory testing at the CDC, so likely represents a false-positive.

*Patient 2*: A 65-year-old man presented in June 2014 with three weeks of frontal headaches, intermittent photophobia, and episodes of garbled speech and clumsiness. He subsequently developed visual hallucinations and left hand numbness. CSF analysis showed elevated protein and mild lymphocytic pleocytosis, and MRI showed scattered, nonspecific, FLAIR hyperintense foci in the cerebral parenchyma. His medical history was notable for hemochromatosis and seronegative symmetric polyarthritis. He spent time outdoors on Cape Cod. He was treated empirically for meningitis but returned ten days later with recurrent confusion and clumsiness. He was given steroids and improved, but returned three weeks later for ongoing motor deficits resulting in a fall. His mental status deteriorated and he became unresponsive. Brain biopsy showed scattered perivascular subarachnoid granulomata with negative stains for microorganisms, and he was provisionally diagnosed with CNS vasculitis/granulomatous angiitis; he had moderate improvement on cyclophosphamide. Ultimately, serum and CSF were positive by JCV IgM ELISA with PRNT results of 1:1280 and 1:8, respectively. The patient had ongoing waxing and waning spasticity, unsteadiness, and depression.

*Patient 3*: A 31-year-old man developed fatigue in August 2014, followed by two weeks of headaches, drenching sweats without fever, forgetfulness, and mental fog. He was treated empirically with doxycycline. Two months later, he was referred to the infectious disease clinic for persistent symptoms. His physical exam was unremarkable and he did not undergo LP. His medical history was notable only for hereditary elliptocytosis. He reported frequent exposure to ticks and farm animals; his occupation required him to work outdoors in northeast Massachusetts. Serum was positive by JCV IgM ELISA with a PRNT of 1:1280. La Crosse virus (LACV) was also positive by ELISA, but with PRNT 1:160 and less epidemiological risk of exposure, this was considered to represent cross-reactivity. The patient’s symptoms resolved by ten weeks after onset.

*Patient 4*: A 63-year-old man presented to clinic in September 2014 with three days of fever, chills, and malaise. He had nausea, diarrhea, and a diffuse headache. He was febrile to 101.6 F, with no focal neurological deficits. He reported spending extensive time outdoors in both northeastern Massachusetts and southern Maine. He did not undergo brain imaging or LP. He was treated empirically with doxycycline but Lyme ELISA was negative. Serum JCV IgM ELISA was positive with a PRNT of 1:320. The patient’s symptoms completely resolved within two months.

**Figure 1.**
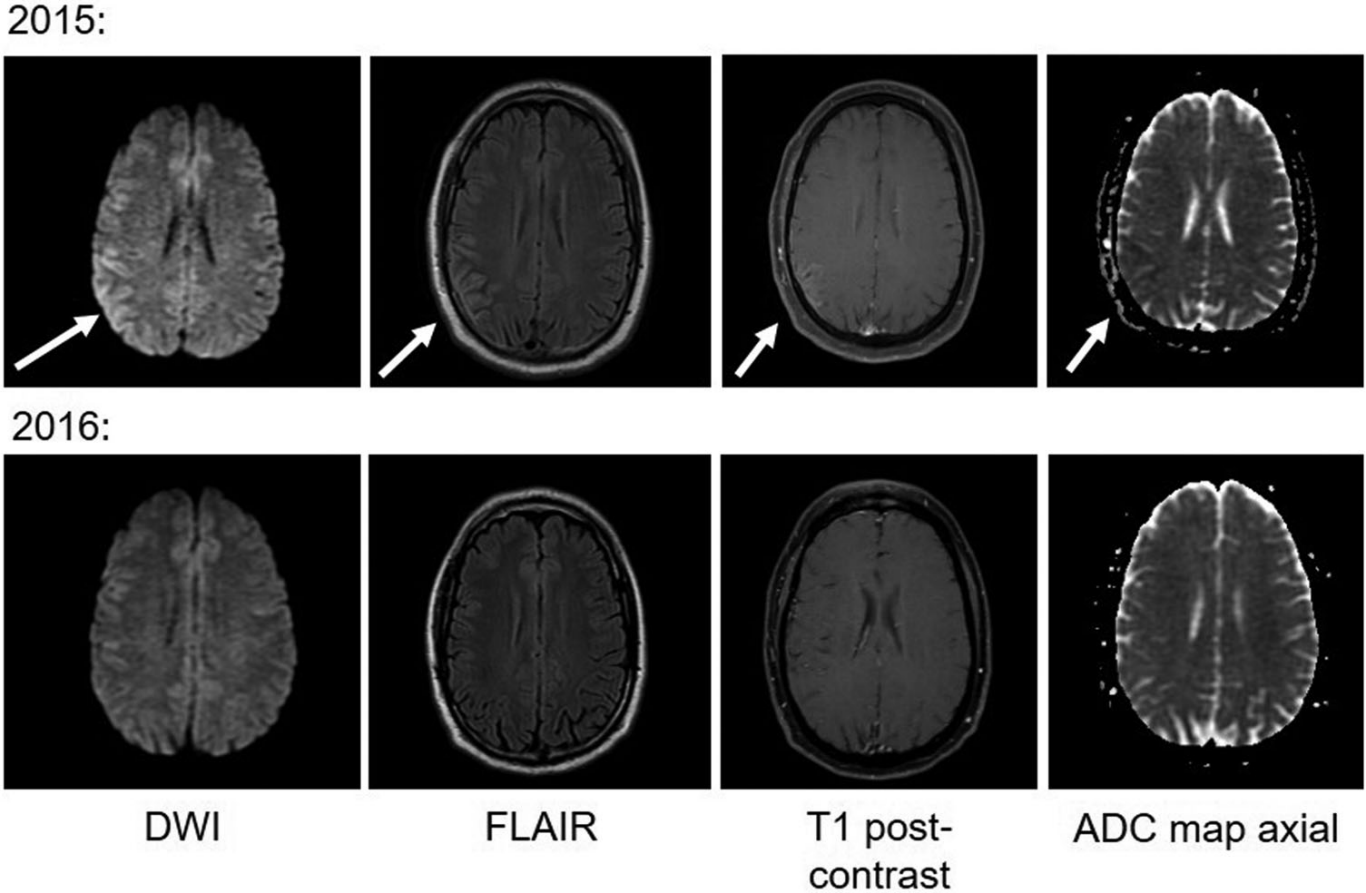
Brain MRI findings from Patient 5. Arrows highlight areas of abnormality in the right parietal cortex, which resolved over 14 months.

*Patient 5*: A 36-year-old man presented with three weeks of headache and three days of altered sensory perception in June 2015. He was afebrile, with pressured speech but no focal neurological findings. MRI showed gyriform enhancement with FLAIR hyperintensity and restricted diffusion in the right parietal cortex (Figure 1). CSF analysis showed elevated protein and lymphocytic pleocytosis; CSF and plasma glucose were elevated, and he was diagnosed with diabetes mellitus. He spent significant time outdoors including recently in Maine and New York, but had not noticed tick and mosquito bites. Serum was positive by JCV IgM ELISA with a PRNT of 1:160. The patient had persistent headache and fatigue over the subsequent year. Repeat MRI 14 months later was normal (Figure 1).

*Patient 6*: A 68-year-old woman developed subjective fever, headache, and generalized arthralgia in July 2016. She treated herself with doxycycline for two days, with improvement. However, for the next several months, she had severe fatigue, myalgia, and intermittent headaches. Lyme IgM and IgG Western blots were positive, and she was treated with doxycycline without further improvement. Brain MRI was normal. LP, performed five months after symptom onset, had no abnormalities. The patient reported numerous mosquito bites from eastern Massachusetts. Serum was positive by JCV IgM ELISA with a PRNT of 1:80. The patient received no further treatment, and was lost to follow up.

*Patient 7*: A 57-year-old man presented with six days of increasing headache, fever to 102.9 F, and confusion in November 2016. He was confused and agitated on presentation. He had no new focal neurological deficits but had persistent left superior quadrantopsia from a recent occipital stroke; brain MRI was compatible with this and showed no new abnormalities. CSF analysis showed a lymphocytic pleocytosis with elevated protein. The patient had a history of rheumatoid arthritis (treated with prednisone 10 mg per day and prior methotrexate) and bicuspid aortic valve with ascending aortic aneurysm, recently repaired. He lived in central Massachusetts on a farm and spent substantial time outdoors. An engorged *Ixodes scapularis* tick was discovered during his hospitalization, and he was treated for presumed Lyme meningoencephalitis. A serum sample sent on admission was positive by JCV IgM ELISA with PRNT of 1:2560. The patient slowly improved, with persistent headache that was attributed to migraine. Two months after admission, he had continued improvement in memory and cognition and was working but had ongoing fatigue.

*Patient 8*: A 28-year-old man presented with two days of headache and confusion in August 2017. He had no fever or abnormalities on examination. Head CT was normal, and LP was not done. The patient had persistent dull headache, neck stiffness, and fatigue 12 days later, and was evaluated in an infectious disease clinic. MRI of the brain was normal. LP, performed two and a half weeks after symptom onset, was unremarkable. The patient was otherwise healthy. He spent significant time outdoors, noted mosquito bites, and had recently travelled to Maine. Serum was positive for JCV IgM ELISA with a PRNT of 1:160. Arbovirus testing was also positive for Saint Louis encephalitis virus (SLEV) IgM with PRNT ≥1:10, however this finding was not replicated on confirmatory testing at the CDC, so likely represents a false-positive. At six-month follow-up, the patient continued to have intermittent dull headaches.

*Patient 9*: A 40-year-old man with recent history of back pain experienced a sudden loss of consciousness in September 2017. He was afebrile, disoriented to time and place, and reported severe left frontal and eye pain. A seizure was considered likely. LP and MRI were both unremarkable. Serum was positive for JCV IgM ELISA with a PRNT of 1:160. No specific mosquito exposure was reported. His hospital stay was significant for persistent retrograde amnesia. At one month post-discharge, his memory was gradually returning, and he reportedly felt well but had not returned to work.

### Molecular testing of clinical samples for JCV RNA

Many of the patients described here experienced a delay in diagnosis, including empiric treatment for more common infections, before arbovirus serology was requested and results returned, highlighting the need for rapid clinical diagnostic tests. We therefore tested the feasibility of molecular detection of JCV RNA using the few residual clinical samples available. From Patient 2, an archived serum sample from approximately 1 month after symptom onset was available, and was negative by JCV RT-qPCR. From Patient 4, an archived serum sample from approximately 3 days after symptom onset was available, and was also negative by RT-qPCR. Patient 7 had been enrolled in a study of pathogen identification by metagenomic sequencing; no JCV reads were identified in CSF obtained approximately two days after symptom onset, or in plasma obtained approximately one week after symptom onset (Piantadosi *et al*., in preparation). Although based upon a limited number of samples, these results do not support routine testing for JCV RNA in immunocompetent patients, similar to other neuroinvasive arboviruses such as West Nile virus [17,19].

### Mosquito screening, sequencing, and analysis

Pools of *O. canadensis* collected between 2012 and 2016 were screened by JCV RT-qPCR (*N* = 359). One pool (Pool 10) was positive, with a concentration of 273 copies/µl (mean Ct 25.7). The other 358 pools were negative for JCV by RT-qPCR.

**Figure 2.**
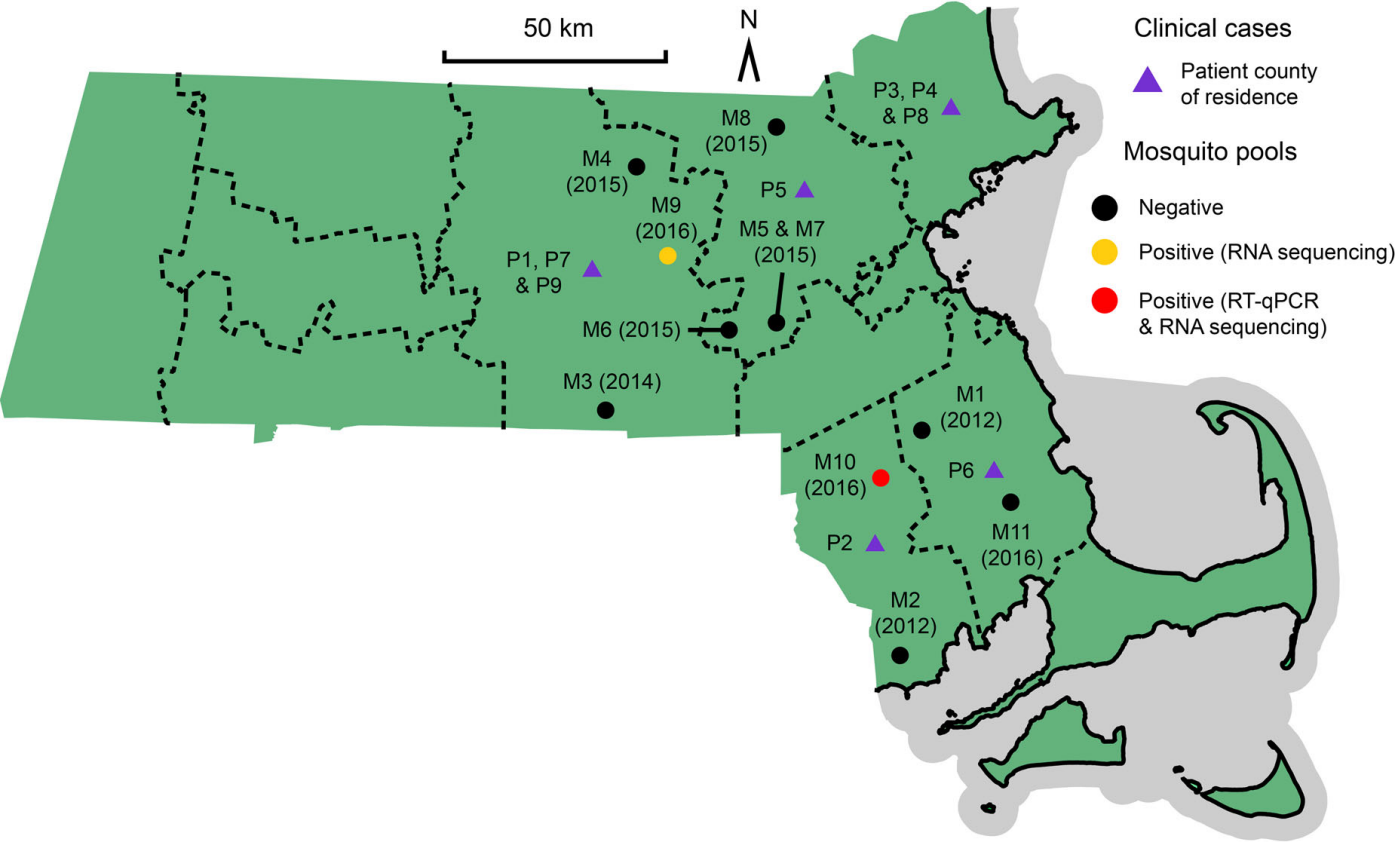
Map of Massachusetts indicating the county of residence for each of the patients described (purple triangles). Also shown are the locations of the eleven mosquito pools that underwent both RT-qPCR and RNA sequencing, two of which were JCV positive (red and yellow circles). The complete JCV genome JCV/16/MA/01S was derived from M10.

RNA sequencing was performed for the positive pool, along with nine other pools whose RT-qPCR results were negative but close to the Ct threshold in ≥1 well (Table 3, Figure 2), as well as one negative control pool. In order to distinguish low-level positive samples from between-sample contamination, samples that contained more than one JCV read underwent construction of an independent sequencing library starting from RNA, and reads were BLASTn searched in order to ascertain which JCV strain they belonged to. Ultimately, Pool 10 was confirmed to have high-level JCV content by both RT-qPCR and RNA sequencing, while Pool 9 was negative by RT-qPCR (with low-level amplification in 3 wells at mean Ct 37.1) but positive by sequencing.

**Table 3.** RT-qPCR and sequencing results for 11 mosquito pools.

	JCV RT-qPCR results			No. JCV sequencing reads		No. total sequencing reads	
Sample	Positive	Mean Ct	Mean copies/uL	Library 1	Library 2	Library 1	Library 2
No template controls	2/75	35.5	NA	NA	NA	NA	NA
Pool 1	1/3	36.6	0.8	0	NA	2,780,216	NA
Pool 2	1/3	35.6	0.4	0	NA	3,436,372	NA
Pool 3	0/3	NA	NA	0	NA	4,640,644	NA
Pool 4	3/3	39.2	<0.1	0	NA	2,796,224	NA
Pool 5	3/3	34.1	0.4	0	NA	2,714,312	NA
Pool 6	2/3	41	<0.1	0	NA	2,390,196	NA
Pool 7	3/3	36.1	0.1	0	NA	1,687,562	NA
Pool 8	2/3	35.7	0.2	2	0	3,694,624	4,782,330
Pool 9	3/3	37.1	0.4	6	4	3,725,980	5,849,716
Pool 10 (screening)	3/3	25.7	272.9	390	>1000	1,597,522	7,602,802
Pool 10 (deep sequencing)				>1500	>4000	13,829,206	32,622,656
Pool 11	1/3	33.4	2.7	0	NA	1,971,412	NA

Notes: Pool 10 was positive by RT-qPCR (all replicates positive, below the Ct of no template controls), while Pool 3 was a negative control and the remaining pools were negative for JCV by RT-qPCR, but showed some amplification close to the threshold. Based on sequencing results from both libraries combined, Pools 9 and 10 were confirmed as positive for JCV. Pool 10 underwent deeper sequencing in order to assemble a full JCV genome.

**Figure 3.**
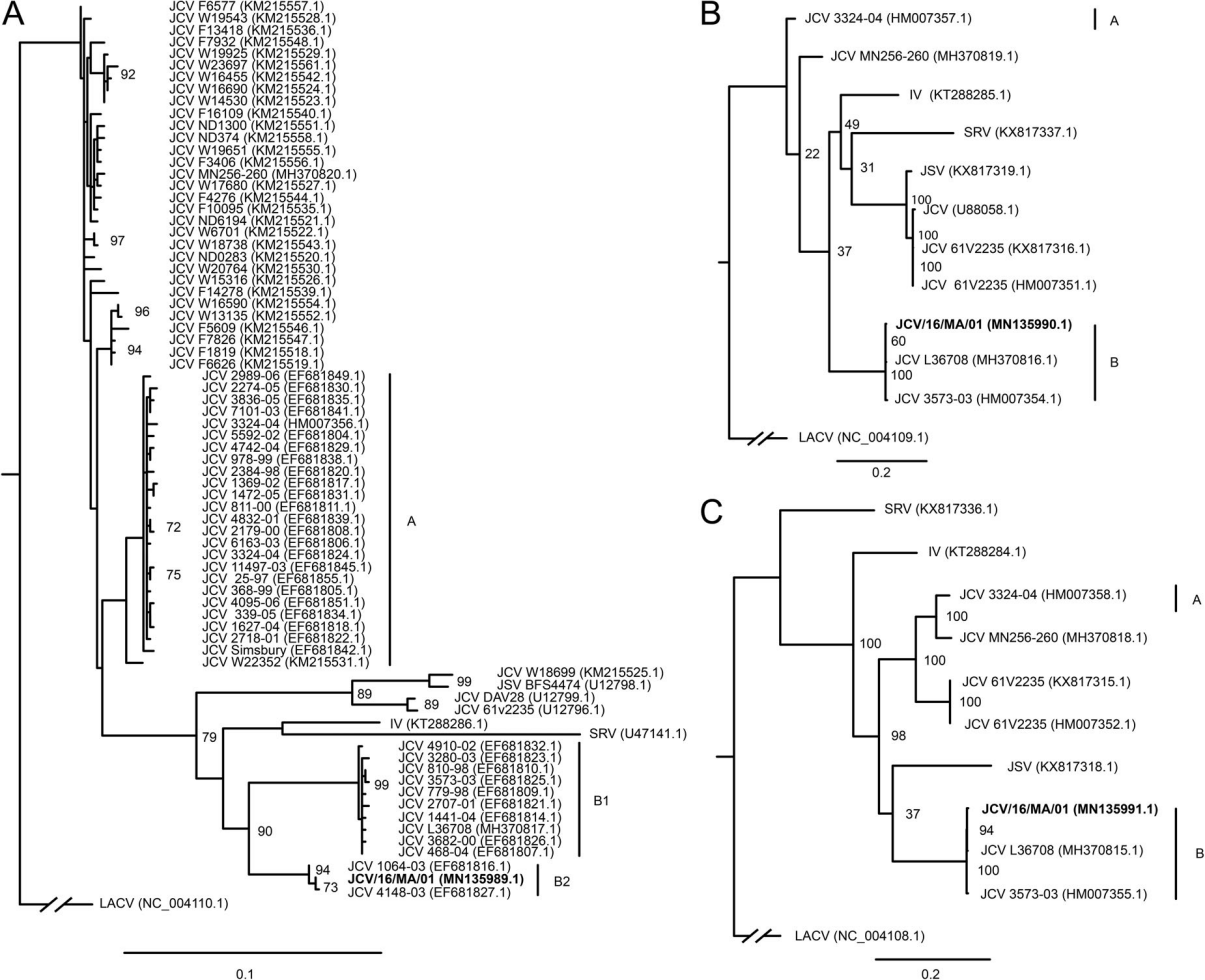
Maximum likelihood phylogenetic tree of JCV/16/MA/01 S segment (panel A), M segment (panel B), and L segment (panel C) compared to previously published reference sequences. Scale bars refer to nucleotide substitutions per site.

JCV reads from Pool 10 were successfully assembled into the complete coding sequences of the S, M, and L genome segments with mean coverage depths of 122X, 64X, and 33X respectively (accessions MN135989-91). This genome, hereafter referred to as JCV/16/MA/01, was assembled from a pool of *O. canadensis* mosquitoes trapped in Bristol County, Massachusetts, in June 2016. Phylogenetic analysis of the JCV S segment recapitulated the A, B1, and B2 clades described from mosquitoes in Connecticut [31] (Figure 3a). The novel genome JCV/16/MA/01 clustered within the rarely isolated B2 clade. It was most closely related (100% nucleotide identity in the S segment) to a JCV isolate from Tolland County, Connecticut collected in 2000 (EF681828.1). It also shared 95–99% identity across all three segments to an early JCV isolate collected in Simsbury County, Connecticut in 1966 (MH370815-7). Phylogenetic analysis of the few available M and L segments produced a similar topology, separating the A and B lineages (Figure 3b,c).

## Discussion

The neuroinvasive arbovirus JCV has been increasingly recognized as a public health concern [32]. The patients described here reflect the elevated number of cases identified in Massachusetts after introduction of JCV IgM ELISA screening at the MDPH, as part of a CDC-led project involving several states.

We describe nine cases of neuroinvasive JCV infection in Massachusetts between 2013 and 2017. The patients in our series were similar to previously described patients, most from a U.S.-wide series of 31 cases from 2000 to 2013 and a description of 15 patients with neuroinvasive disease in Wisconsin from 2011 to 2016 [1,4–8,33]. Patients usually presented between June and August and were generally healthy, but some had mild immune suppression such as diabetes mellitus or prednisone use. Most were active outdoors and had noted mosquito bites. Interestingly, eight of the nine patients in our series were men, consistent with prior observations that men are more frequently diagnosed with arboviral infections [8]. Indeed, of the 175 reported cases of JCV between 2013 and 2018, 67% of patients were male [4–9].

Similar to prior cases, common symptoms among our patients included headache and altered sensorium; one patient in our series had seizure, which has also been reported in a previously published case [14]. Only two had documented fever, though several had chills or sweats. There were no notable peripheral laboratory abnormalities, and inflammatory markers were normal when checked. Most patients had unremarkable imaging. CSF studies showed mild to moderate pleocytosis with lymphocytic predominance, consistent with prior reports [2,3,14]. Most patients were diagnosed by serology from serum rather than CSF, as in prior cases [1]. Most patients had a months-long recovery, including persistent fatigue, cognitive difficulties, and headache.

Our cases highlight several challenges associated with JCV diagnostics. Clinically, JCV is indistinguishable from other neuroinvasive arboviruses and can resemble more common infections such as Lyme meningitis, so confirming the diagnosis relies upon laboratory testing. Diagnostic testing for JCV is currently performed by the CDC’s Arboviral Diseases Branch and a limited number of state public health laboratories. Serology remains the gold standard; although PCR is faster than serology, it is unreliable for many arboviruses due to short and/or limited viremia. In our assessment of molecular tests for JCV, we did not detect JCV RNA by RT-qPCR from serum samples from two patients, one of which was obtained only three days after symptom onset. Similarly, we did not detect JCV by RNA sequencing from acute serum and CSF from a third patient (Piantadosi *et al.*, in preparation). While these were residual samples that may have been degraded by freeze/thaw cycles, our findings are consistent with prior results; JCV has never been isolated from human samples, and one study demonstrated no detectable viremia in experimentally infected rhesus macaques, despite seroconversion [34]. In general for orthobunyaviruses, it has been challenging to grow virus [35] or detect viral RNA in human samples; the few exceptions to this have included the detection of RNA from LACV in brain tissue [36], Oropouche virus in leukocytes [37], and Cache Valley virus in the CSF of an immunocompromised patient [38]. Overall, our results suggest that viral RNA detection techniques are unlikely to be useful for routine JCV diagnostics, likely because of low-level or short viremia. An important exception to this is immunosuppressed patients, who may have a longer period of detectable viremia, as well as negative serology (Solomon *et al.*, in preparation).

Illustrating an important challenge of serology-based testing, three patients in our series had positive IgM for LACV. Like JCV, this is a California serogroup Orthobunyavirus, but is not found in New England. Prior studies have noted LACV cross-reactivity in up to 23% of patients with JCV [1], and it is suspected that many patients historically diagnosed with LACV may have instead had JCV [39]. An additional important observation is the clinical suspicion and empiric treatment for Lyme disease in several of our patients, even with negative Lyme serology. This suggests that JCV should be considered in patients with suspected Lyme CNS infection but negative testing for Lyme disease.

The primary vector for JCV in Massachusetts is unknown; we hypothesized that *O. canadensis* is important given its predominance in Connecticut [21]. Among 359 mosquito pools collected between 2012 and 2016, two (0.6%) were positive for JCV by metagenomic sequencing. This is similar to the 0.5% rate observed in *O. canadensis* pools in Connecticut from 1997 to 2006 [21], suggesting a stably low prevalence in New England. However, *O. canadensis* may not be the primary vector for JCV. Unlike most arboviruses, JCV does not have a strict vector range [21,40], and it is possible that human infection is derived from multiple species [23,41–43]. Notably, a single mosquito pool contained a high concentration of JCV, while another had only a low number of reads detected by sequencing, suggesting a very low viremia. JCV may generally persist at low levels in mosquitoes, similar to other orthobunyaviruses in arthropod vectors [40,44].

The full coding sequence of JCV/16/MA/01 was successfully assembled from a pool of mosquitoes trapped in Bristol County, Massachusetts, in June 2016, the first JCV genome from the state. This JCV genome is also the first sequenced directly from the primary mosquito sample, and was therefore unaffected by passaging in cell culture. JCV/16/MA/01 belongs to the rarely isolated B2 lineage [31]. Interestingly, although prior JCV sequencing has indicated geographic structure across Connecticut, our sequence (from southeast Massachusetts) was identical in the S segment to a JCV isolate from Tolland County, central Connecticut, isolated in 2000 [31] (4473-00, accession EF681828.1), and very similar to a JCV isolate from Litchfield County, western Connecticut, isolated in 2003 (1064-03, accession EF681816.1). This suggests that there may be more widespread circulation of JCV strains than previously recognized. Additionally, it is notable that the JCV/16/MA/01 sequences were so similar to strains isolated more than a decade apart. Very few nucleotide changes have been observed in JCV lineage A across 40 years, however little is known about the other lineages, and this is the first lineage B2 virus sampled since 2003. The high conservation of JCV/16/MA/01 compared to JCV isolates from Connecticut over the last 60 years thus supports the slow rate of evolutionary change that has been previously described [31]. This, and the relatively low vector prevalence are surprising given the increasing number of reported infections with this understudied and morbid virus; further investigation is warranted to explore this discrepancy.

## Supplementary Material

Supplemental Material

## Data Availability

Assembled genome sequences are available under GenBank accessions MN135989-91. Sequence reads are available under SRA BioProject PRJNA622688.
